# Effect of Lankford Coefficients on Springback Behavior during Deep Drawing of Stainless Steel Cylinders

**DOI:** 10.3390/ma16124321

**Published:** 2023-06-11

**Authors:** Fei Wu, Yihao Hong, Zhengrong Zhang, Chun Huang, Zhenrong Huang

**Affiliations:** 1 School of Material and Energy, Guangdong University of Technology, Guangzhou 510006, China; julie.wu@gdut.edu.cn (F.W.); hyhlztt@163.com (Y.H.);; 2 Guangdong Provincial Key Laboratory of Metal Forming and Forging Equipment Technology, Foshan 528300, China

**Keywords:** lankford coefficient, deep drawing, springback, stainless steel cylinder

## Abstract

Accurate prediction of springback is increasingly required during deep-drawing formation of anisotropic stainless steel sheets. The anisotropy of sheet thickness direction is very important for predicting the springback and final shape of a workpiece. The effect of Lankford coefficients (r_00_, r_45_, r_90_) with different angles on springback was investigated using numerical simulation and experiments. The results show that the Lankford coefficients with different angles each have a different influence on springback. The diameter of the straight wall of the cylinder along the 45-degree direction decreased after springback, and showed a concave valley shape. The Lankford coefficient r_90_ had the greatest effect on the bottom ground springback, followed by r_45_ and then r_00_. A correlation was established between the springback of workpiece and Lankford coefficients. The experimental springback values were obtained by using a coordinate-measuring machine and showed good agreement with the numerical simulation results.

## 1. Introduction

As an important metal-forming process, sheet metal stamping is widely applied in the modern industry [1,2]. Springback is an inevitable physical phenomenon during the metal sheet-forming process [3,4,5]. The influence of springback on the accuracy and tolerance of a dimension is remarkable. The traditional trial-and-error and empirical methods for weakening springback and obtaining height-precision parts are time-consuming and expensive. The occurrence of defects, such as wrinkling, cracking, and springback, during sheet formation can be predicted with numerical simulations [6,7,8,9]. However, the predictions of springback and the final shape of the workpieces have a low accuracy rate because of the strong plastic anisotropy in the thickness direction.

A lot of research has been carried out in order to understand the influence of material properties and process parameters on springback behavior. Huang [10] analyzed the effects of different process parameters on springback during the stamping process using finite element numerical simulations. Minh [11] also used finite element simulation to analyze the effects of various factors—such as the blank holder force, friction coefficient, and blank thickness—on the springback of high-strength steel. Based on the numerical simulation results, it was evident that the blank holder force and blank thickness were the main factors affecting springback. Hashem and Roohi [12] utilized a numerical simulation to determine the effect of die and punch profile radii, as well as blank holder force on the springback and thinning percentage in the deep-drawing process of the cylindrical parts. The results show that an increased springback is observed due to an increased punch radius, and punch corner radius has been identified as the most significant effect on springback. Lajarin [13] found the blank holder force to be the most influential parameter for the springback of high-strength steel, followed by the die radius and friction conditions. Starman [14] proposed a numerical method to optimize the blank shape and tool geometry in a 3D sheet-metal-forming operation, with the effects of sheet-metal edge geometry and springback after forming and trimming being considered throughout the optimization process. Huang et al. [15] studied the defect behavior during the stamping of thin-walled semicircular shells with a bending angle via an analytical model, experiments, and a finite element (FE) simulation. The springback decreased with the increasing of blank holder force and decreasing of stamping speed. Aydın et al. [16] investigated the formability and springback behavior of dualphase (DP600) and high-strength low-alloy (HSLA) sheets bonded with laser-beam welding. The results show that springback behavior changes depending on the die angle and holding time. Where the die angle is up to 45°, the springback angle increased by 10.4%. When holding time was increased by 10-s, the springback angle decreased by 21.2%, on average. Saito et al. [17] carried out a springback experiment of 980 MPa on high-strength steel sheets with V-shaped and U-shaped bending at temperatures ranging from room temperature to 973 K. The amount of springback decreased with the temperature rise, especially at temperatures above 573 K. Springback was much reduced at lower forming speeds. The influence of stress relaxation on springback was investigated using the V-shaped bending springback test and viscoplastic stress analysis. Chang et al. [18] studied the bending springback of medium-Mn steel, a third-generation automobile steel, under different working conditions through experiment and simulation, and they also analyzed the influences of rolling direction, bending angle, and punch fillet radius on springback. The effect of the rolling direction on the springback angle was negligible. The bending angle had a positive effect on the springback angle, while the punch fillet radius had a negative effect.

During recent decades, many researchers have explored the influence of anisotropy on springback and its prediction. Ragai et al. [19] provided an experimental and computational study of springback during draw-bending of stainless steel 410. The effect of several parameters such as blank holding force, lubrication, and anisotropy on springback were discussed. Parsa et al. [20] studied the springback of hyperboloid sheet metal formation theoretically, numerically, and experimentally. Emphatically, they analyzed the influence of thickness and curvature radius on springback. The experimental results showed that the influence of material anisotropy on the forming springback of hyperboloid sheet metal is related to material parameters. Gomes et al. [21] investigated the variation in springback in high-strength steels due to material anisotropy. They analyzed and compared material models based on different yield criteria using the geometry of a standard U-shape. The results showed a discrepancy between springback predicted by the various material models and the variations in springback from 0- and 90-degree material orientation. Leu and Zhuang [22] developed a simplified approach by considering the thickness ratio, normal anisotropy, and the strain-hardening exponent to estimate the springback angle in the vee bending process for high-strength steel sheets. The numerical simulation showed that the springback ratio increased as normal anisotropy increased or as the thickness ratio and the strain-hardening exponent decreased. Verma and Haldar [23] investigated the effect of anisotropy on springback for the benchmark problem in Numisheet-2005. An analytical model was developed to cross-check the prediction from the finite element analysis. Both of the models predicted that higher anisotropy leads to more springback. Lee et al. [24] investigated the influences of the anisotropy and friction models for high-strength steel sheets during U-draw/bending and suggested the optimum selection of the models for springback simulations. Jung et al. [25] developed an elastoplastic material constitutive model with anisotropic evolution, which they then applied to a U-bending process. By comparing the measured springback angle with the predicted springback angle, they showed that the model could accurately predict the springback angle in different directions.

Stainless steel sheets are widely used in modern industry, and springback during the stamping process is a common problem. Various process parameters can affect springback during the deep-drawing process of anisotropic stainless steel sheets. Although many scholars have conducted investigations on the springback problem during sheet metal formation using the finite element numerical simulation method and experimental methods, the research on springback of anisotropic sheet metal is still mostly confined to the forming of V/U-shaped parts, and there is little research on other common forming parts, for example, cylindrical cups. The influence of Lankford coefficients with different angles on springback during the cylinder deep-drawing process has not been clearly researched. 

In this paper, a cylinder deep-drawing process with anisotropic stainless steel sheets was simulated based on the Barlat–Lian 1989 anisotropy yield criterion [26] by using Dynaform 5.9 software, and was used to predict springback. The Taguchi and ANOVA techniques were utilized to establish the correlation between springback at different angles from the rolling direction and Lankford coefficients (r_00_, r_45_, r_90_) of 304 stainless steel. The ANOVA showed that the Lankford coefficient had a significant effect on springback. This research shows that each Lankford coefficient has an obvious influence on springback in diffident angles from the rolling direction by using the experimental and numerical simulation.

## 2. Finite Element Simulation (FEM) Analysis

### 2.1. FEM Simulation Procedure

**Figure 1:**
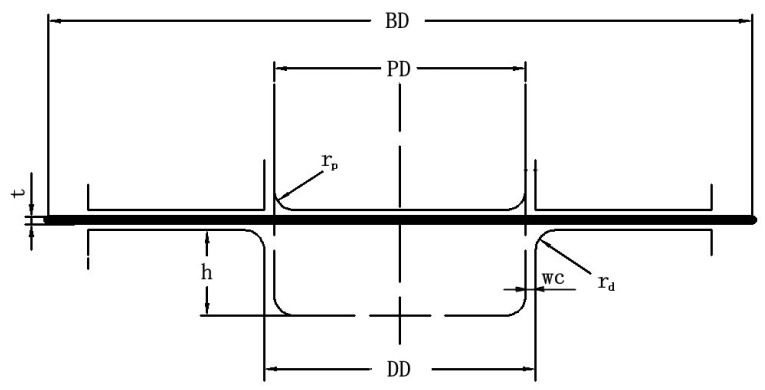
Geometry of drawing die.

**Figure 2:**
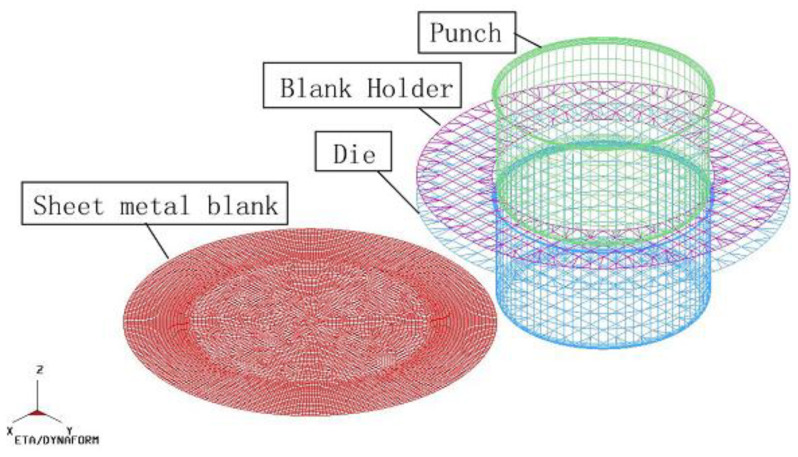
Model in FEM.

In this paper, the finite element numerical simulation was carried out on Dynaform. Dynaform software is a special piece of software jointly developed by ETA and LSTC for numerical simulation of sheet metal formation. It is a combination of LS-DYNA solver and ETA/FEMB front and back processor, and it is one of the most popular CAE tools for sheet metal formation and die design. Figure 1 shows the cylinder deep-drawing die; the actual object of the model is thecooking pot. The dimensions of the blank, die, punch, and blank holder are given in Table 1. One quarter of the 3D numerical model can be applied to the FEM model. The simulations require a large amount of computational time if they are not simplified, but they can provide a greater degree of precision. In this paper, a complete 3D numerical model was used. The 3D numerical model is shown in Figure 2. Table 2 shows the Lankford coefficients of metal sheets in different rolling directions with two horizontal factors set. The other mechanical properties of the materials were imported from the materials library in Dynaform. The punch and die were set as rigid, and the velocity of the punch was set at 2000 mm/s. The friction coefficient between the tools and the blank were set to 0.125. The contact-one-way surface-to-surface mode was employed to determine the friction type, and the adaptive meshing method was adopted to mesh the geometry model [27]. The full integrated planar shell was used, and the element type was defined as the time-efficient full-order integral Belytschko–Tsay shell element. This allowed for the adoption of four-point integration in order to avoid the appearance of “hourglassing” mode. A dynamic explicit algorithm was used to calculate the forming process. The implicit algorithm was applied to calculate the springback process. 

**Figure 3:**
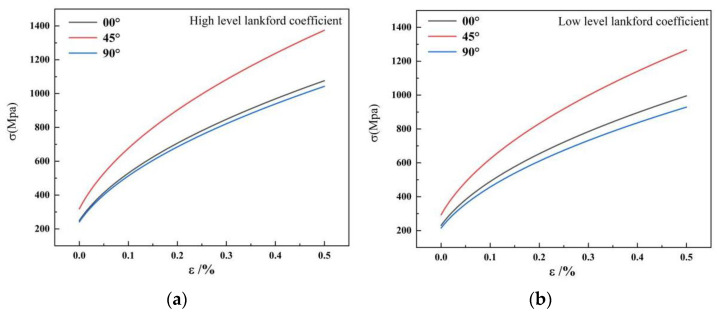
The stress–strain curves in different angles to the rolling direction: (**a**) high-level Lankford coefficient material hardening curve and (**b**) low-level Lankford coefficient material hardening curve.

The material was modeled as an elastic–plastic material. The anisotropic characteristic was described by the Barlat–Lian 1989 anisotropic yield criterion [28]. The Barlat–Lian 1989 anisotropic yield criterion and the Hosford series’ yield criterion were used to analyze the plastic flow law of the drawing process [29,30,31]. Three stress–strain curves were obtained from the tensile test for the model material, as shown in Figure 3. The different curves were determined according to the ratio of the Lankford coefficients in each direction of the actual material.

### 2.2. Taguchi Technique

The Taguchi technique was applied to the design scheme of the numerical simulation [32]. The two levels of the three-parameter orthogonal design, considering interactions (2^7^), are presented in Table 3. The springback of different angles from the rolling direction was the process response. In order to understand the influence of Lankford coefficients, the ANOVA technique was applied to illustrate the degree of significance of each Lankford coefficient, including interactions.

### 2.3. Measurement Set-Up

**Figure 4:**
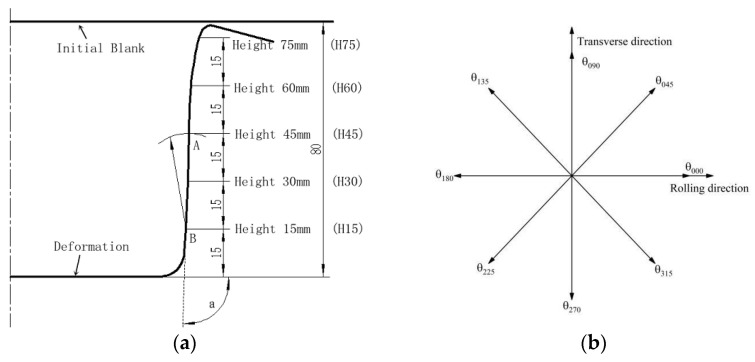
The Schematic diagram of the measurement position: (**a**) deformation and measurement locations and (**b**) diagrammatic sketch of angles from the rolling direction.

Figure 4 shows the typical shape characteristics and measurement locations of the cylindrical cup. After formation, the workpiece was measured using CMM. Angles (α) were measured every 45 degrees from the rolling direction, and diameters were measured every 15 mm in the five sections along the height. A diagrammatic sketch of angles from the rolling direction is shown in Figure 4.

### 2.4. Formation Analysis

**Figure 5:**
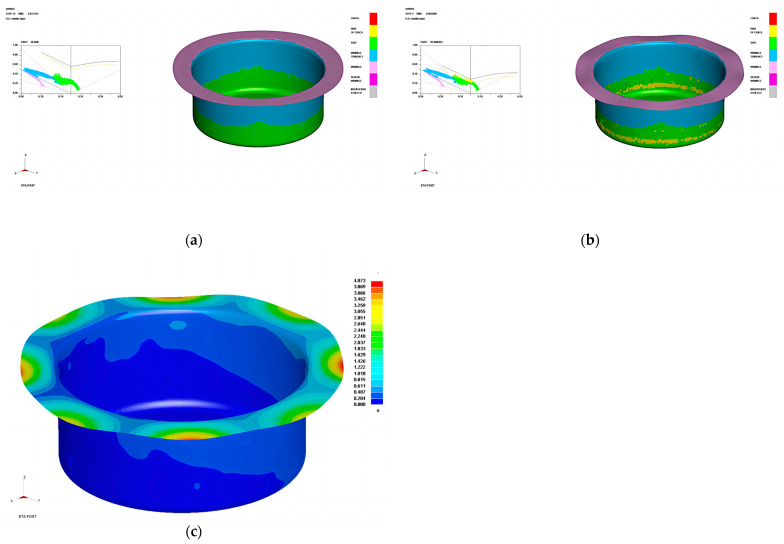
Forming limit diagram of the cylindrical cup and the cloud diagram of springback: (**a**) limit diagram of the cylindrical cup, (**b**) Limit diagram of the cylindrical cup after springback, and (**c**) cloud map of the cylindrical cup after springback.

The forming limit diagram (FLD) and thickness change diagram can intuitively show the dynamic drawing process of the sheet metal and predict the formation of defects, such as cracking and wrinkling, and the thickness distribution of the sheet metal [33]. Figure 5a shows the forming limit diagram of the cylindrical cup after deep-drawing formation, Figure 5b shows the forming limit diagram of the cylindrical cup after springback, and Figure 5c is the cloud diagram of springback change in the cylindrical cup after springback calculation. It can be seen that the cylindrical cup fluctuates after springback with different degrees in the flange. The springback is apparent at 0°, 45°, and 90° positions, which shows a cyclical trend of first decreasing and then increasing along the rolling direction. The straight wall of the cylinder also showed uneven springback.

**Figure 6:**
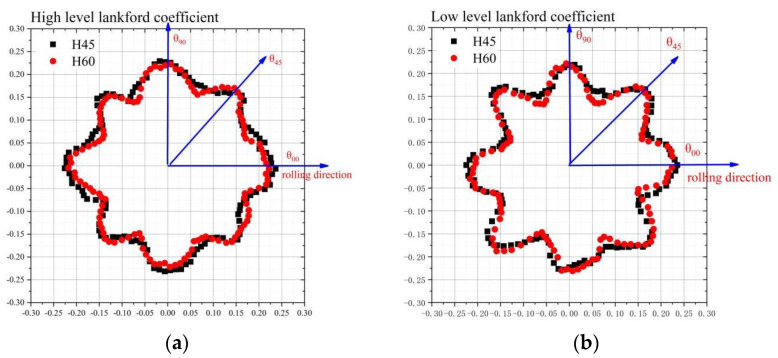
The springback difference point cloud of the cross-section of the cylindrical cup: (**a**) high–level Lankford coefficient material and (**b**) low–level Lankford coefficient material.

Due to the uneven springback deformation of the straight wall of the cylindrical cup, the sections with heights of 45 mm and 60 mm were selected for measurement, and 120 coordinate points were measured for each section. The difference between coordinate values of data points before and after springback was calculated. The cross-section difference point cloud diagrams are shown in Figure 6. The co-ordinates only represent the position of data points on the section of the cylindrical drawing section. The distance between each point and the origin represents the springback value. It can be seen that the springback difference between the two heights is similar, and is in the range of 0.150–25 mm. Within the angle of 0–45° from the rolling direction, the springback difference firstly decreases, and then it increases. At the position of the maximum plastic strain value of r_45_, that is, at the positions 45°, 135°, 225°, and 315° from the rolling direction, the springback difference of the cylinder drawing part reaches its maximum value.

### 2.5. Stress–Strain Analysis

**Figure 7:**
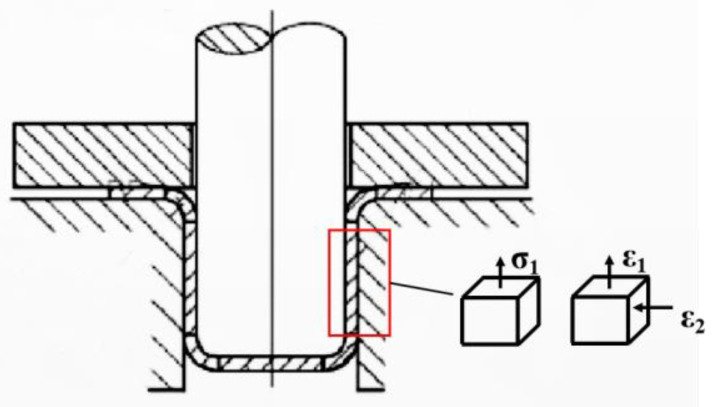
The stress and strain state of straight wall area.

The straight wall of the cylindrical cup is an area of force transmission during deep drawing, and no more plastic deformation occurs. The straight wall experiences a single axial tensile stress. There is a small amount of axial elongation and deformation. The state of stress and strain during deep drawing is shown in Figure 7. The first principal stress and strain of the model of the straight wall model’s middle layer was extracted to analyze the reasons for uneven springback.

**Figure 8:**
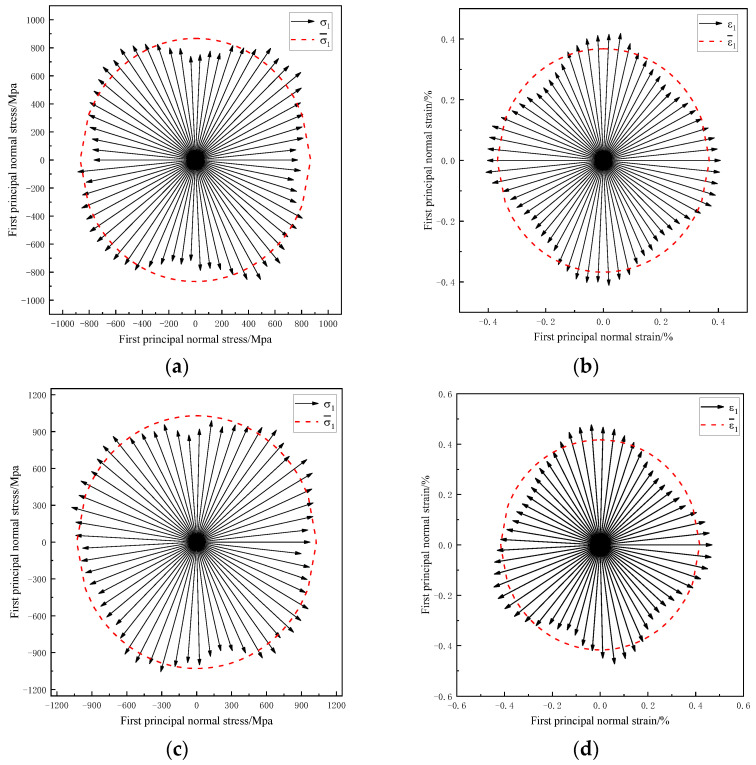
Stress and strain states on sections with different heights: (**a**) stress values at the 45 mm height position, (**b**) strain values at the 45 mm height position, (**c**) stress values at the 60 mm height position, and (**d**) strain values at the 60 mm height position.

The stress–strain analysis diagrams of cylindrical deep drawing at the heights of 45 mm and 60 mm are shown in Figure 8. The stress–strain data of 60 points on the circumference of the straight wall were extracted, and the red circle represents the average stress–strain value of all points. It can be seen that, at the height of 45 and 60 mm, the first principal stress was greater than the other two directions at the position of 45° from the rolling direction, while the first principal strain was smaller than the other two directions.

The stress–strain values for the three rolling directions of 0°, 45°, and 90° were compared and analyzed, and the results are shown in Table 4. The stress at 45° at the height of 45 mm is 23% higher, and the stress at 45° at the height of 60 mm is 19.37% higher than that of the other rolling directions. This is because the hardening curves are for different rolling directions. The value of the hardening curve at 45° from the rolling direction was larger, and the stress value required during deep drawing was larger. The strain in the 45° direction was smaller and contained more elastic stress in the deformation process, resulting in greater springback deformation after unloading.

### 2.6. Boundary Inflow Analysis

**Figure 9:**
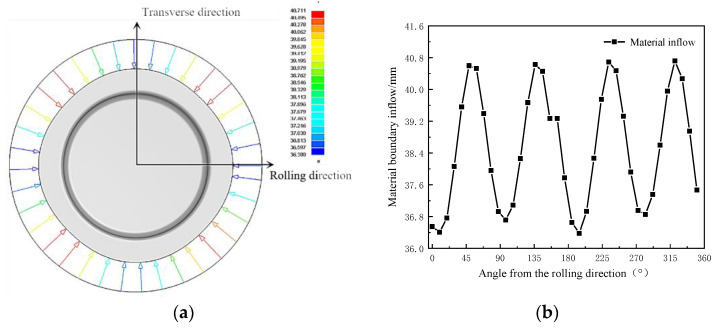
The boundary inflow of material and the trend of material inflow: (**a**) schematic diagram of the material boundary inflow and (**b**) numerical change diagram.

The diagram of inflow of the cylindrical cup boundary material is shown in Figure 9. It shows a cyclical trend of first increasing and then decreasing between 0° and 90° from the rolling direction. At the positions 45°, 135°, 215°, and 315°, there was a larger inflow, and the maximum value was 40.76 mm. The Lankford coefficients in the 45° direction were greater than those for the 0° and 90° directions. When the Lankford coefficients were large, the deformation resistance of the flange of the metal sheet was reduced, and the material flowed more easily. The flow stress value in the 45° direction was large, so the inflow of material was larger. The flow stress in the 0° and 90° directions was smaller, elongation deformation was easier, and the inflow was smaller. This may be one of the reasons for the greater springback difference in this direction.

The sheet firstly underwent elastic deformation, and then plastic deformation occurred after the stress exceeded the flow stress during the deformation process. After unloading, the internal stress was redistributed, and then springback occurred. The deformation and plastic deformation of the cylindrical cup drawing in the 45° direction was less severe than the other two directions. The circumferential stress in the 45° direction was relatively large, resulting in larger springback deformation at this position. The diameter of the cylinder after springback in the 45° direction was smaller, showing a greater springback difference.

## 3. Experimental Procedures

### 3.1. Experimental Set-Up

**Figure 10:**
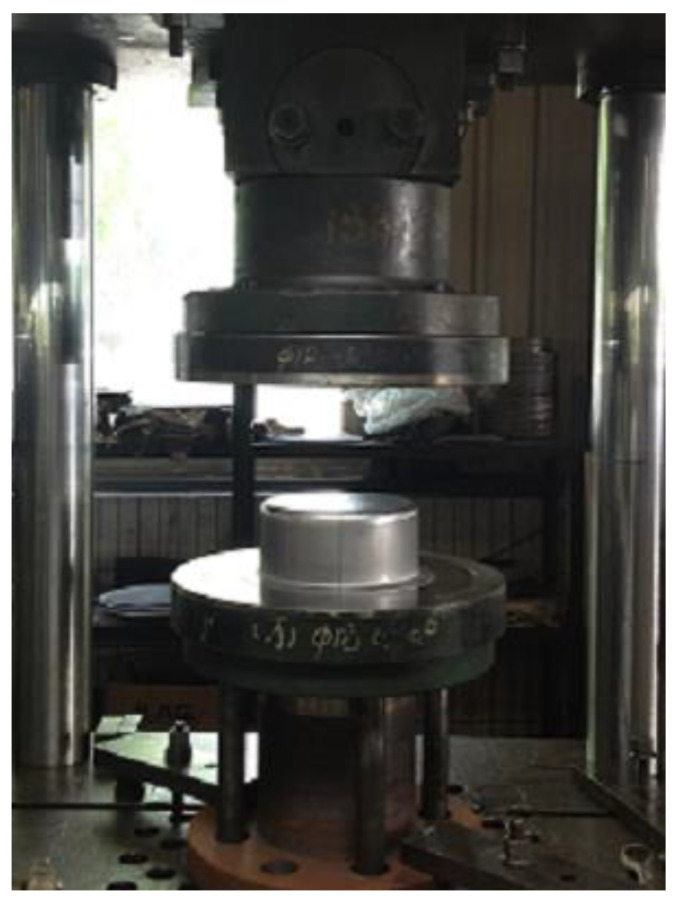
Die assembly for experimental drawing.

Two different stainless steel sheets with the same thickness were selected for the experimental test. Based on the previous experimental tests [34,35], strong anisotropic properties were present in the two materials. Lankford coefficients of r_00_, r_45_, and r_90_ are listed in Table 2. For cylinder deep drawing, circular blanks with a diameter of 315 mm and a thickness of 0.6 mm were prepared. Figure 10 shows the drawing die designed and fabricated based on the simulation model.

### 3.2. Experimental Results

**Figure 11:**
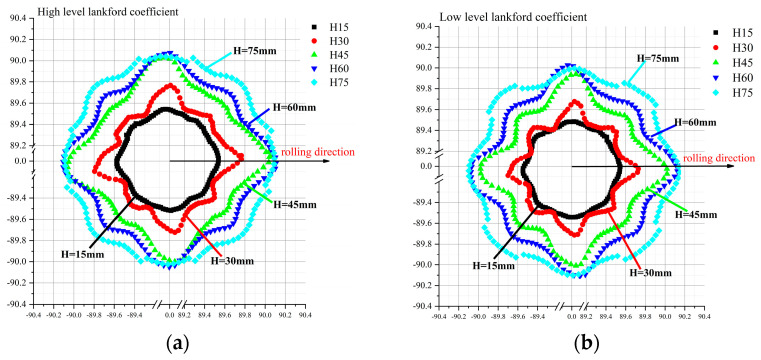

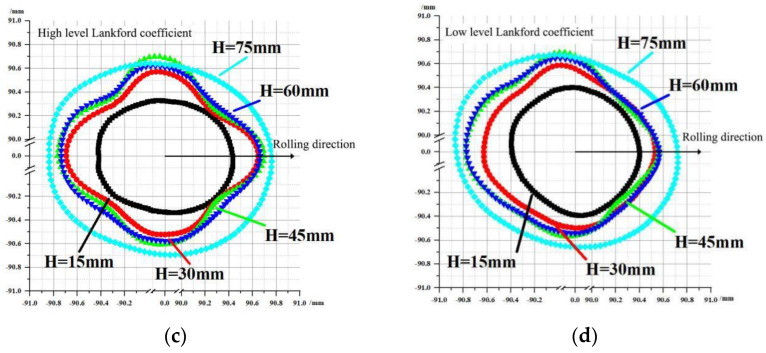
Average vertical wall shell’s dimension of the experiments and simulations: (**a**) high-level Lankford coefficient material simulation results, (**b**) low-level Lankford coefficient material simulation results, (**c**) high-level Lankford coefficient material experimental results, and (**d**) low-level Lankford coefficient material experimental results. Note: Black: H = 15 mm Red: H = 30 mm Green: H = 45 mm Blue: H = 60 mm Light Blue: H = 75 mm.

After calculating the weighted average of the co-ordinates of two types of stainless steel workpieces at different heights, the radius values at different angles were obtained, as shown in Figure 11. The average values of the cross-section point cloud can be compared at the height of 30 mm, 45 mm, and 60 mm. The valley shape of the depression was clearer and more obvious in the material with the larger Lankford coefficients at the positions of 45°, 135°, 225°, and 315°. At the height of 15 mm near the bottom of the cylinder, the low-Lankford-coefficient material showed a more rounded cross-section. The high-Lankford-coefficient material showed a less rounded cross-section after drawing, and it was close to an ellipse along the long axis of the rolling direction and the short axis perpendicular to the rolling direction. At a height of 75 mm near the flange, the sections of both kinds of stainless steel showed an elliptical shape after deep drawing and springback. The experimental results are in good agreement with the FEM simulation results.

Table 5, Table 6, Table 7 and Table 8 compare the radius values along the three rolling directions at five section heights obtained from numerical simulation and experiments. It can be seen that the difference in radius between different rolling directions is more obvious in the simulation, and it was the largest at the heights of 45 mm and 60 mm. In the material with high-level Lankford coefficients, the differences reached 0.433 mm and 0.318 mm, respectively. In the material with low-level Lankford coefficients, the differences reached 0.387 mm and 0.32 mm, respectively. The experimental difference between radii in different rolling directions was close to the simulation result in the material with high-level Lankford coefficients. The difference at the heights of 30 mm and 45 mm reached 0.158 mm and 0.204 mm, respectively. The experimental radius difference between different rolling directions in material with low-level Lankford coefficients was small, reaching 0.127 mm and 0.08 mm at the heights of 45 mm and 60 mm, respectively.

**Figure 12:**
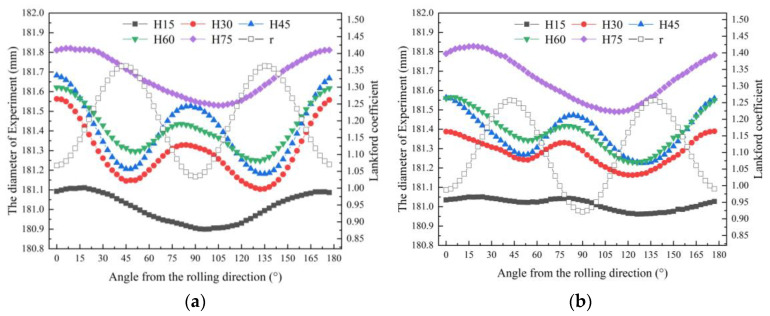
Diameter of different sections of experimental measurements: (**a**) the material with high-level Lankford coefficients and (**b**) the material with low-level Lankford coefficients.

Figure 12 shows experimental measurements of diameter at five sections along the height. They have a similar trend. The section at the height of 15 mm was close to the radius of the punch nose. The section at the height of 75 mm was similar to the radius of the die shoulder. The fillet radius had a great influence on the workpiece diameter. The cross-section of the cylinder after deep drawing showed an oval shape after springback. The diameters at the height of 30, 45, and 60 mm showed a similar trend. The results showed that the r_45_ Lankford coefficient is the maximum value. In addition, as the Lankford coefficient increased, the diameter decreased.

## 4. Results and Discussions

### 4.1. Application of ANOVA

Table 9 shows the results of springback prediction by FEM simulation, which shows that the springback of every angle from the rolling direction is without symmetrical characteristics.

To investigate the degree of significance of the Lankford coefficients, the ANOVA technique was used to analyze the springback. The mean overall value $S/N\left(\bar{S/N}\right)$ is expressed as Equation (1), where *k* is the number of simulations. The range of two levels (*SR_j_*) is shown in Equation (2). The sum of squares owing to the variations of the overall mean (*SS*) and the mean of the Lankford coefficients with interactions (*SS_j_*) are expressed as Equations (3) and (4), respectively. The percentage values (*%p-Value_j_*) were calculated using Equation (5), which is generally applied when measuring the degree of significance of each Lankford coefficient [36].

(1) $$\bar{S/N}=\frac{1}{8}\overset{8}{\underset{k=1}{\sum }}{\left(S/N\right)}_{k}$$

(2) $$SR_{j}=\overset{7}{\underset{j=1}{\sum }}\left({\left(S/N\right)}_{1j}-{\left(S/N\right)}_{2j}\right)$$

(3) $$SS=\overset{8}{\underset{i=1}{\sum }}{\left({\left(S/N\right)}_{ij}-\bar{S/N}\right)}^{2}$$

(4) $$SS_{j}=\overset{7}{\underset{j=1}{\sum }}{\left({\left(S/N\right)}_{ij}-\bar{S/N}\right)}^{2}$$

(5) $$\%p-Value_{j}=\left(\frac{SS_{j}}{SS}\times 100\right)$$

### 4.2. Effects of Process Parameters on Springback

The results of the range analysis and variance analysis are shown in Table 10. It is revealed that the influence of Lankford coefficients on springback is different at different angles. The source of r_00_ had a critical effect on the springback at θ_000_ from the rolling direction, the r_45_ is the key factor causing springback at θ_045_ and θ_315_, and r_90_ is key at θ_135_, θ_180_, and θ_225_. Furthermore, r_45_ × r_00_ has significant values for springback at θ_090_ because of interactions with Lankford coefficients. Meanwhile, r_90_ × r_45_ is the key factor of springback at θ_270_. The measurement error also easily affected the range analyses, and so the ANOVA technique was used to analyze the springback. The ANOVA results shown in Table 9 correspond well with the range analysis results. Based on these analysis results, it has been found that the interactions of Lankford coefficients at different angles from the rolling direction have a clear effect on the springback.

**Figure 13:**
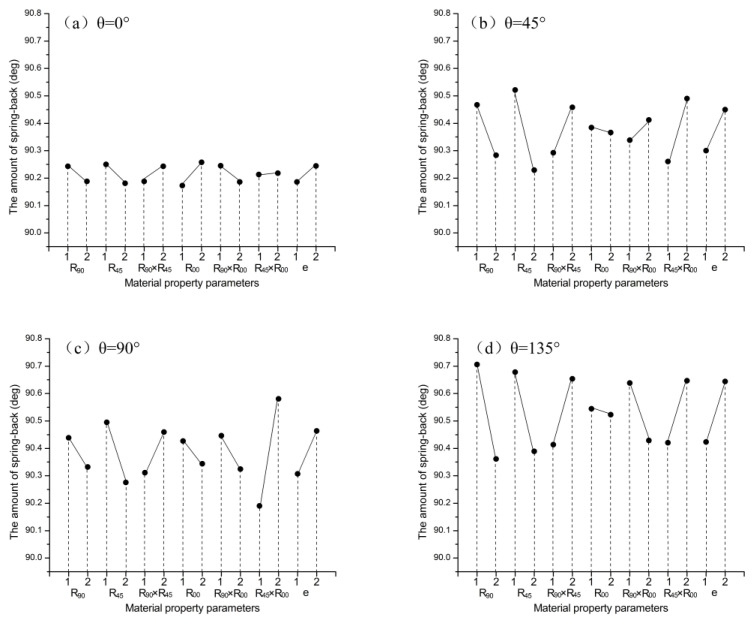

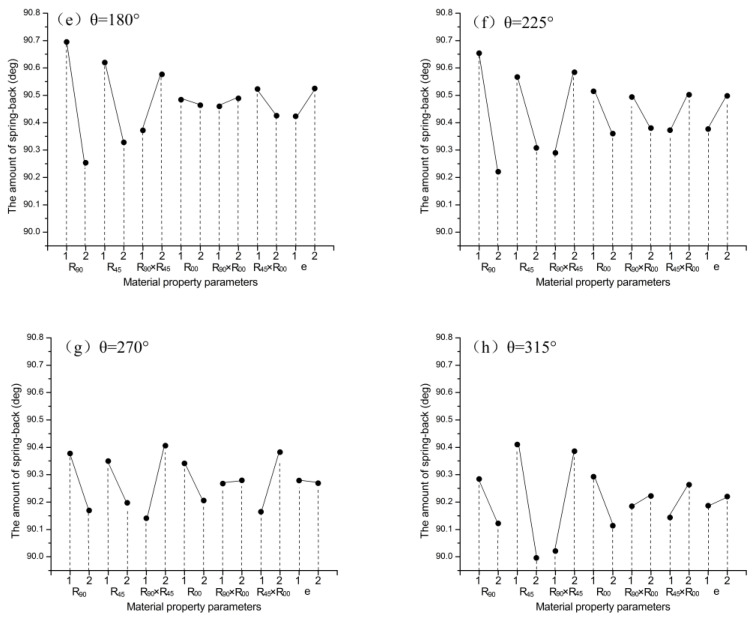
The sensitivity analysis of the effect of Lankford coefficients on springback.

Figure 13a–h shows the sensitivity analysis of the effect of Lankford coefficients on springback. When the interactions of the Lankford coefficients were not considered, the amount of springback decreased with r_90_ and r_45_ at all angles, and the amount of springback decreased with increasing r_00_, except at the angle of θ_000_. When considering the interactions, the amount of springback increased with increasing r_90_ × r_45_. When the r_45_ and r_90_ increased simultaneously, the interaction of r_90_ × r_45_ hindered springback and caused it to decrease. The amount of springback increased with increasing r_45_ × r_00_, except at the angle of θ_180_. The amount of springback decreased with increasing r_90_ × r_00_, except at the angles of θ_045_ and θ_180_. The results show that the influences of the Lankford coefficient on springback at different angles are interrelated and interact with each other.

### 4.3. Comparing the FEM Simulation and Experimental Results

**Figure 14:**
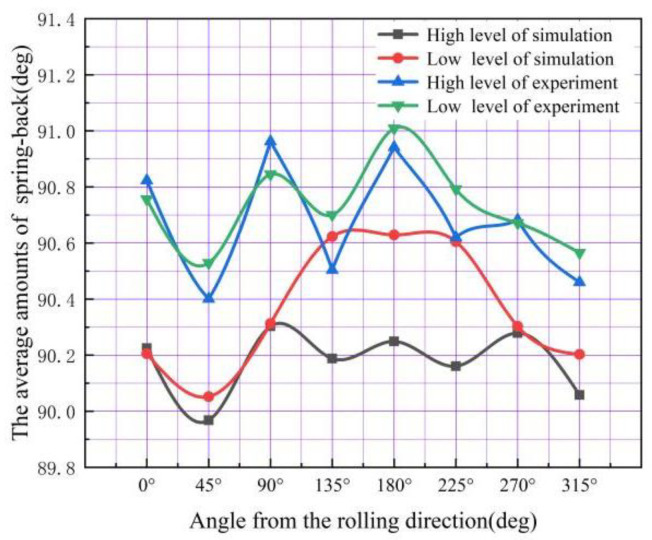
Comparison of the average of experimental results and the FEM simulation results.

The experimental results of two kinds of 304 stainless steel were measured using CMM, as shown in Table 11. The No. 1 and No. 8 FEM simulation results are shown in Table 12. Figure 14 shows the comparison of an average bottom fillet of the FEM simulation and experimental results at different angles from the rolling direction. The material with high-level Lankford coefficients had a larger amount of springback at 0, 90, and 270 degrees from the rolling direction. The experimental results have good agreement with the FEM simulation results, with the bottom fillet showing a similar trend. The springback of the cylinder bottom fillet occurred along the rolling direction, and there was an increasing trend with every 45° decrease.

**Figure 15:**
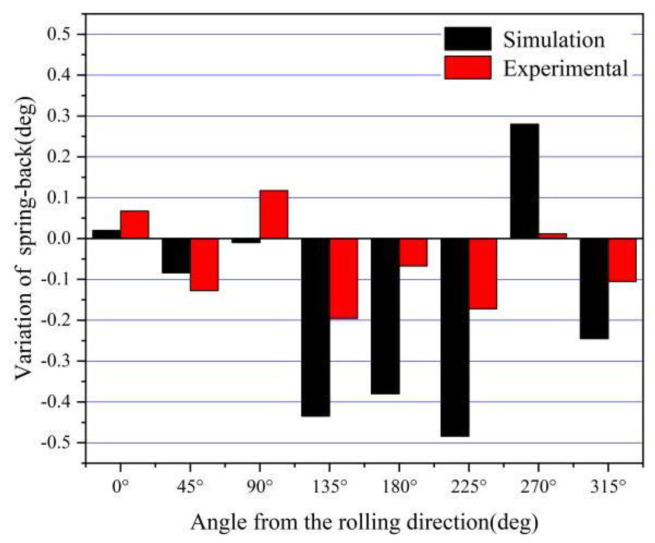
Comparison of the variation of angles between the FEM simulation results and the experimental results.

The comparison between the bottom fillets of materials with high- and low-level Lankford coefficients is shown in Figure 15. The experimental results have good agreement with the FEM simulation results, showing a similar trend in springback. The amount of the bottom fillet decreased with an increase in the Lankford coefficient at all locations, except for 0, 90, and 270 degrees. The trend is more apparent in the FEM simulation results.

## 5. Conclusions

The influence of Lankford coefficients on stainless steel cylindrical cups was investigated using both experiments and numerical simulation. The conclusions are as follows:

The simulation and experimental results show that the Lankford coefficients had an obvious effect on the cross-section diameter. The flow velocity of the blank was different because of the anisotropy of the metal sheet, which makes the stress–strain values accumulate in different directions during the deep-drawing process, and finally causes a clear difference in springback. The simulated springback value for the straight wall was between 0.15 mm and 0.25 mm. The maximum springback value was at the position of 45° from the rolling direction. Specifically, the diameters at different height sections were related to the Lankford coefficients at different angles from the rolling direction, which were characterized by a concave valley in the 45 degree direction of the straight wall. The radius difference between the 45 degree rolling direction and the other two directions at each section height was between 0.1 mm and 0.3 mm.

The ANOVA results illustrated the influence of Lankford coefficients on the springback of the bottom fillet. The Lankford coefficient has different levels of effects on springback depending on the angle from the rolling direction. The 90-degree angle had the greatest influence, followed by the 45-degree, with 0 degrees having the least influence. The experimental results showed a similar trend to the simulation results. In addition, the springback of the bottom fillet decreased with the increasing overall Lankford coefficients.

The combination of the FEM simulation, the ANOVA technique, and the experimental study of the cylinder deep-drawing process is an effective method for studying the influence of Lankford coefficients on springback and predicting the final shape with high precision.

In this paper, the study of the effect of Lankford coefficients on springback of a cylindrical cup during the deep-drawing process remained at the macroscopic stage, and further analysis on the microscopic aspects was not carried out. The mechanism by which metal anisotropy influences the springback of a cylinder needs to be explored further. Although some characteristic rules regarding springback and cylindrical cup properties during deep drawing were obtained in this study, the analysis of the specific degree of influence of Lankford coefficients on springback properties is still in the preliminary stage. Therefore, the quantitative analysis of the influence of Lankford coefficients on the springback of the cylindrical cup during deep drawing will remain the focus of future research. Based on the previous research on the cylindrical cup, the springback of large complex thin-walled parts in deep drawing will be further explored.

## Figures and Tables

**Table 1:** Basic geometrical parameters.

Parameter	Dimension in mm
blank size diameter (BD)	315
blank thickness (t)	0.6
punch diameter (PD)	180
punch nose radius (rp)	8
die shoulder radius (rd)	4
die diameter (DD)	181.32
radial clearance between punch and die (wc)	0.66
height of drawing (h)	80

**Table 2:** Test factors and their levels.

	Factors	r		
Level		r_00_	r_45_	r_90_
Low-level		0.99	1.26	0.92
High-level		1.07	1.36	1.03

**Table 3:** Experimental design of orthogonal considering interactions (2^7^) for FEM simulation.

Simulation No.	Factors						
	r_90_	r_45_	r_90_ × r_45_	r_00_	r_90_ × r_00_	r_45_ × r_00_	Error
1	Low	Low	Low	Low	Low	Low	Low
2	Low	Low	Low	High	High	High	High
3	Low	High	High	Low	Low	High	High
4	Low	High	High	High	High	Low	Low
5	High	Low	High	Low	High	Low	High
6	High	Low	High	High	Low	High	Low
7	High	High	Low	Low	High	High	Low
8	High	High	Low	High	Low	Low	High

**Table 4:** The stress and strain in three directions at the heights of 45 mm and 60 mm of high-level Lankford coefficient material.

	Direction	0°	45°	90°	Difference (Max–Min)
Height					
H45					
Stress/Mpa		770.475	947.854	782.773	177.379 (23%)
Strain		0.407	0.349	0.396	0.058
H60					
Stress/Mpa		973.37	1162	997.662	188.63 (19.37%)
Strain		0.468	0.368	0.463	0.1

**Table 5:** The radius values of the simulations with high-level Lankford coefficients on the three directions.

	Direction	0°/mm	45°/mm	90°/mm	Difference /mm
Height/mm					
H15		89.561	89.492	89.537	0.068
H30		89.987	89.740	89.983	0.246
H45		90.506	90.072	90.486	0.434
H60		90.617	90.299	90.592	0.318
H75		90.599	90.575	90.517	0.024

**Table 6:** The radius values of the simulations with low-level Lankford coefficients on the three directions.

	Direction	0°/mm	45°/mm	90°/mm	Difference /mm
Height/mm					
H15		89.541	89.490	89.495	0.051
H30		89.884	89.710	89.883	0.174
H45		90.428	90.041	90.404	0.387
H60		90.603	90.283	90.557	0.320
H75		90.652	90.585	90.515	0.067

**Table 7:** The radius values of the experiments with high-level Lankford coefficients on the three directions.

	Direction	0°/mm	45°/mm	90°/mm	Difference /mm
Height/mm					
H15		90.546	90.506	90.452	−0.007
H30		90.781	90.563	90.660	0.158
H45		90.841	90.597	90.760	0.204
H60		90.811	90.642	90.708	0.118
H75		90.905	90.837	90.775	0.003

**Table 8:** The radius values of the experiments with low-level Lankford coefficients on the three directions.

	Direction	0°/mm	45°/mm	90°/mm	Difference /mm
Height/mm					
H15		90.517	90.498	90.516	0.019
H30		90.694	90.610	90.645	0.060
H45		90.781	90.628	90.729	0.127
H60		90.780	90.654	90.697	0.085
H75		90.895	90.826	90.767	0.005

**Table 9:** The results of springback prediction by FEM simulation.

	The Amount of Springback/(°)								
	θ_000_	θ_045_	θ_090_	θ_135_	θ_180_	θ_225_	θ_270_	θ_315_	Difference (Max–Min)
1	90.205	90.052	90.313	90.623	90.629	90.645	89.999	90.303	0.44
2	90.294	90.748	90.645	90.838	90.617	90.729	90.363	90.315	0.544
3	90.255	90.566	90.779	91.021	90.445	90.931	90.601	90.407	0.766
4	90.217	90.242	90.027	90.342	90.654	90.412	90.268	90.112	0.627
5	90.205	90.518	90.379	90.529	90.626	90.514	90.338	90.577	0.421
6	90.295	90.507	90.652	90.723	90.175	90.481	90.418	90.447	0.428
7	90.027	90.14	90.246	90.006	90.069	89.968	90.148	89.884	0.362
8	90.225	89.968	90.303	90.188	90.249	90.161	90.279	90.058	0.257
average	90.215	90.375	90.385	90.534	90.433	90.480	90.302	90.263	0.319

**Table 10:** Results of range analysis and variance analysis.

	Range Analysis	Variance Analysis (The Key Factor and Contribution Value)
θ_000_	r_00_	r_00_(28.79%), r_45_(18.94%)
θ_045_	r_45_	r_45_(37.53%), r_45_ × r_00_(23.21%)
θ_090_	r_45_ × r_00_	r_45_ × r_00_(54.46%), r_45_(17.11%)
θ_135_	r_90_	r_90_(29.35%), r_45_(20.66%)
θ_180_	r_90_	r_90_(48.00%), r_45_ × r_00_(23.13%)
θ_225_	r_90_	r_90_(47.89%), r_45_(15.19%)
θ_270_	r_90_ × r_45_	r_90_ × r_45_(38.81%), r_45_ × r_00_(23.17%)
θ_315_	r_45_	r_45_(47.64%), r_90_ × r_45_(33.00%)

**Table 11:** Average experimental results of two kinds of stainless steel.

Material	The Amount of Springback/(°)								
	θ_000_	θ_045_	θ_090_	θ_135_	θ_180_	θ_225_	θ_270_	θ_315_	Difference (Max–Min)
High–level Lankford coefficient	90.823	90.401	90.962	90.504	90.942	90.620	90.683	90.460	0.561
Low–level Lankford coefficient	90.756	90.529	90.845	90.700	91.009	90.792	90.671	90.565	0.48
Difference (Max–Min)	0.067	−0.128	0.117	−0.196	−0.389	−0.172	0.012	−0.105	

**Table 12:** FEM simulation results of springback.

Simulation No.	The Amount of Springback/(°)								
	θ_000_	θ_045_	θ_090_	θ_135_	θ_180_	θ_225_	θ_270_	θ_315_	Difference (Max–Min)
1	90.205	90.052	90.313	90.623	90.629	90.645	89.999	90.303	0.44
8	90.225	89.968	90.303	90.188	90.249	90.161	90.279	90.058	0.257
Difference (Max–Min)	0.02	−0.084	−0.01	−0.435	−0.38	−0.484	0.28	−0.245	

## Data Availability

The data presented in this study are available on request from the corresponding author. The data are not publicly available due to privacy.
